# Detection of Cytosine Methylation in Ancient DNA from Five Native American Populations Using Bisulfite Sequencing

**DOI:** 10.1371/journal.pone.0125344

**Published:** 2015-05-27

**Authors:** Rick W. A. Smith, Cara Monroe, Deborah A. Bolnick

**Affiliations:** 1 Department of Anthropology, University of Texas at Austin, Austin, Texas, United States of America; 2 Department of Anthropology, Washington State University, Pullman, Washington, United States of America; 3 Department of Anthropology, University of California Santa Barbara, Santa Barbara, California, United States of America; 4 Population Research Center, University of Texas at Austin, Austin, Texas, United States of America; Natural History Museum of Denmark, University of Copenhagen, DENMARK

## Abstract

While cytosine methylation has been widely studied in extant populations, relatively few studies have analyzed methylation in ancient DNA. Most existing studies of epigenetic marks in ancient DNA have inferred patterns of methylation in highly degraded samples using post-mortem damage to cytosines as a proxy for cytosine methylation levels. However, this approach limits the inference of methylation compared with direct bisulfite sequencing, the current gold standard for analyzing cytosine methylation at single nucleotide resolution. In this study, we used direct bisulfite sequencing to assess cytosine methylation in ancient DNA from the skeletal remains of 30 Native Americans ranging in age from approximately 230 to 4500 years before present. Unmethylated cytosines were converted to uracils by treatment with sodium bisulfite, bisulfite products of a CpG-rich retrotransposon were pyrosequenced, and C-to-T ratios were quantified for a single CpG position. We found that cytosine methylation is readily recoverable from most samples, given adequate preservation of endogenous nuclear DNA. In addition, our results indicate that the precision of cytosine methylation estimates is inversely correlated with aDNA preservation, such that samples of low DNA concentration show higher variability in measures of percent methylation than samples of high DNA concentration. In particular, samples in this study with a DNA concentration above 0.015 ng/μL generated the most consistent measures of cytosine methylation. This study presents evidence of cytosine methylation in a large collection of ancient human remains, and indicates that it is possible to analyze epigenetic patterns in ancient populations using direct bisulfite sequencing approaches.

## Introduction

Epigenetic marks comprise a variety of stable, chemical modifications to DNA and its associated proteins that influence chromatin structure and regulate gene expression. These marks designate which genomic segments are available for transcription, providing a means for regulating gene activity without changing the underlying nucleotide sequence [1]. Functionally, epigenetic gene regulation plays a crucial role in development, mediates gene-by-environment interactions, and underlies some complex diseases [2–4].

One widely studied type of epigenetic mark is cytosine methylation. In humans and other mammals, cytosines in CpG dinucleotide contexts are targets for epigenetic regulation via cytosine methylation. Methylated cytosines (most commonly 5-methylcytosine, or 5mC) in CpG dinucleotide contexts are vastly underrepresented in the human genome compared to other nucleotide bases and dinucleotide combinations [5], and are often concentrated in regions of high density, such as CpG islands. Other relatively CpG-rich regions of the genome include retrotransposable elements like Long Interspersed Elements (LINEs) and Short Interspersed Elements (SINEs), which are usually epigenetically inactivated through cytosine methylation to prevent aberrant transposition [6–7].

**Fig 1:**
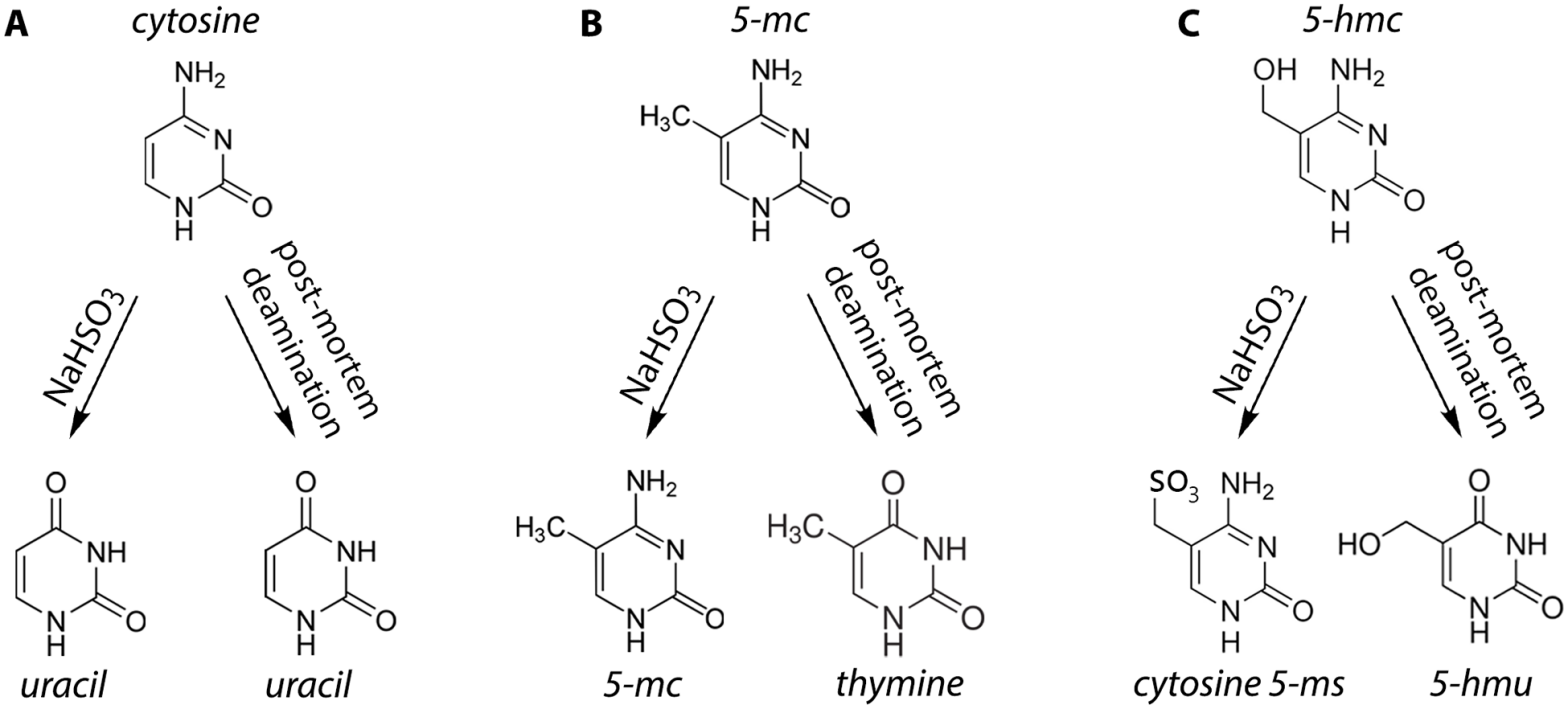
Detecting methylation of cytosine residues and their deamination products. 5-mc: 5-methylcytosine; 5-hmc: 5-hydroxymethylcytosine; 5-ms: 5-methylenesulfonate; 5-hmu: 5-hydroxymethyluracil; NaHSO_3_: Sodium bisulfite. (*A*) Unmethylated cytosines are converted to uracil at high efficiency by bisulfite conversion and at low efficiency by post-mortem deamination. After conversion, no methylation is detected by either bisulfite sequencing or misincorporation analysis. (*B*) Methylated cytosines are unaffected by bisulfite conversion, while post-mortem deamination converts methylated cytosines to thymines. Methylation is detected by the presence of undamaged cytosines in bisulfite sequencing, and by the presence of thymines at damaged positions in misincorporation analysis. (*C*) Hydroxymethylated cytosines are converted to cytosine 5-methelensulfonate by bisulfite conversion, and 5-hydroxymethyluracil by post-mortem deamination. Methylated cytosines are detected at undamaged positions by bisulfite sequencing, but cannot be discriminated from non-hydroxylated methylcytosines using this method. It is currently unclear whether misincorporation analysis will be able to detect methylation in the form of 5-hydroxymethyluracil, but the UDG-endoVIII approach may be able to do so.

While cytosine methylation has been widely studied in extant species, relatively few studies have attempted to analyze epigenetic marks in the DNA of ancient or extinct organisms. Recently, however, several studies have indicated that cytosine methylation can be reconstructed in ancient specimens. Briggs and colleagues [8] found the first evidence for cytosine methylation in aDNA extracted from 43,000-year-old *Mammuthus primigenius* and 38,000-year-old Neanderthal remains. In their study, uracil-DNA-glycosylase (UDG) and endonuclease VIII (endoVIII) were used to repair aDNA extracts to reduce sequencing errors resulting from post-mortem damage. This treatment removed uracils formed by the deamination of unmethylated cytosines, which greatly increased aDNA sequence accuracy by reducing C/G→T/A conversions. However, the researchers observed an incomplete rescue of C/G→T/A misincorporations and found that the unrepaired base misincorporations were concentrated in CpG dinucleotide contexts of nuclear aDNA [8]. This pattern suggests that the methylated status of cytosines underlies the unrepaired fraction of misincorporations because the product of 5mC degradation is thymine, a type of DNA damage not repaired by the UDG-endoVIII approach (Fig 1) [8].

These results demonstrate that post-mortem damage patterns can yield information about *in vivo* methylation status. Accordingly, genome-wide methylation maps have recently been inferred for ancient individuals using two approaches that exploit patterns of cytosine deamination. First, using genomic data produced with the UDG-endoVIII method, C-to-T misincorporation rates were analyzed to reconstruct genome-wide methylation levels in Neanderthal and Denisovan individuals [9]. Second, cytosine methylation has been inferred from patterns of DNA degradation by analyzing biases in DNA library composition that stem from the differential DNA replication capabilities of two polymerases, Phusion and HiFi [10]. While both enzymes are capable of duplicating template molecules containing deaminated 5mCs (thymines), only HiFi is capable of duplicating templates containing deaminated unmethylated cytosines (uracils). Phusion amplified libraries therefore contain fewer templates bearing uracils, and these libraries exhibit reduced C-to-T misincorporations near sequence starts compared to HiFi amplified libraries. Since the remaining fraction of misincorporated thymines in Phusion libraries result from the deamination of methylated cytosines, the relative proportion of misincorporations can be used as a general proxy for cytosine methylation levels. Pedersen and colleagues [10] recently used this method to estimate genome-wide methylation levels in a 4000 year old Saqqaq individual from Greenland.

These studies provided the first estimates of genome-wide methylation levels in aDNA, relying on the removal or underrepresentation of uracils in aDNA libraries and assessments of thymine misincorporations. However, there are important methodological limitations that hinder the ability to precisely reconstruct *in vivo* methylation levels and infer gene expression using these approaches. First, while cytosines across the genome are equally likely to experience deamination [9], deamination is a stochastic process and not all cytosines will undergo degradation. Because methylation can only be detected at positions where methylated cytosines have been damaged, samples must be sufficiently degraded in order to infer methylation levels, and even then, incomplete hydrolysis of cytosines limits the ability to precisely reconstruct methylation levels using these methods.

Second, these approaches depend upon the deamination of 5-methylcytosine (5mC) to thymine to detect methylation, but 5mC is not the only form of cytosine methylation. Another form, 5-hydroxymethylcytosine (5hmC), is converted to 5-hydroxymethyluracil (5hmU) by deamination (Fig 1) [11]. The function of 5hmC is not entirely clear, but it is enriched in certain adult human tissues such as bone marrow and brain, and it is concentrated in gene bodies and regulatory elements where it is associated with transcriptional activation [12–13]. This form of cytosine methylation has not been considered in previous studies of methylation in aDNA, and it is not clear if damage-based assessments of methylation will be accurate when 5hmU (the deamination product of hydroxymethylated cytosines) is present. Because UDG does not usually excise 5hmU [14], the UDG-endoVIII method should not repair most 5hmUs, leaving them to be integrated as thymines during template amplification and sequencing. We surmise, then, that the UDG-endoVIII method may still allow methylation to be inferred where 5hmUs are found. However, it is unknown if the Phusion and HiFi polymerases incorporate 5hmU during library construction, so it is unclear if methods using these polymerases yield accurate inferences about cytosine methylation when 5hmU is present. Thus, while cytosine degradation patterns may be exploited to provide a general picture of cytosine methylation levels, it may be difficult to use them to produce precise measurements of cytosine methylation that constitute a high-resolution methylome typically required for the inference of gene expression.

Currently, the gold standard for generating high resolution assessments of cytosine methylation is bisulfite sequencing. If applicable to aDNA, bisulfite methods could circumvent many of the drawbacks associated with inferring methylation from cytosine deamination patterns. Bisulfite sequencing relies on the conversion of unmethylated cytosines to uracils (sequenced as thymines), where the remaining cytosines detected during sequencing are methylated cytosines resistant to conversion by sodium bisulfite (Fig 1). Importantly, bisulfite methods are highly efficient, converting virtually all unmethylated cytosines, while both methylated and hydroxymethylated cytosines (5mCs and 5hmCs) are resistant to conversion. In ancient DNA, bisulfite sequencing would only fail to detect cytosine methylation in molecules where the methylated cytosine experienced post-mortem damage and was replaced by thymine or 5hmU. For relatively well preserved specimens where few DNA molecules exhibit damage at any particular position, this approach should produce a much more accurate picture of cytosine methylation levels and may be preferable to inferring methylation from cytosine damage. However, for extremely degraded samples with poor aDNA quality, inferring methylation from cytosine damage patterns may be preferable to bisulfite conversion, because extensive damage allows for the computational inference of cytosine methylation, and the absence of bisulfite conversion avoids further aDNA degradation of rare samples.

Using bisulfite sequencing, cytosine methylation has been detected in aDNA recovered from the remains of a single 26,000-year-old *Bison priscus* specimen [15]. In this study, Llamas et al. [15] analyzed four repetitive genomic elements and two single-copy imprinted genes. They compared methylation patterns from the extinct specimen with homologues in contemporary *Bos taurus* to establish the validity of the methylation patterns observed in the aDNA sequences. However, while Llamas and colleagues [15] confirmed that methylation patterns can be evaluated via bisulfite methods in aDNA, they reported methylation results for only one of the six ancient samples tested. It is unclear why the other samples did not yield results, but possible reasons include the destructive action of sodium bisulfite on aDNA, which is already heavily degraded by post-mortem processes. Time since death may also matter, because bisulfite sequencing of ancient Egyptian barley recently showed that genome wide demethylation occurs as a time-dependent diagenetic process [16]. However, we do not yet know how often bisulfite approaches will be effective for detecting cytosine methylation in aDNA, or how time since death, depositional context, or parameters of aDNA quality (such as sample concentration) affect the ability to precisely measure methylation via direct bisulfite sequencing.

In this study, we evaluated whether bisulfite methodologies could be used to detect cytosine methylation in the remains of 30 ancient humans from five archaeological sites in North America, ranging in age from approximately 200 years before present (ybp) to more than 4500 ybp. To determine whether cytosine methylation could be recovered in these remains, a single CpG site in the human-specific LINE-1 (L1Hs) element L1Hs56 (GenBank: AC005908) was targeted. L1Hs56 is monomorphic in humans and transcriptionally inactivated by cytosine methylation in normal, somatic tissues such as bone [17–21]. These characteristics make L1Hs56 an excellent target to assess how frequently methylated cytosines are recoverable in ancient human remains. Because time since death and depositional conditions are known to influence aDNA preservation [16,22], we assessed how cytosine methylation patterns were affected by differences in sample age and geographic locality. We also assessed the effects of aDNA quality by comparing cytosine methylation levels with DNA concentration and the presence of co-extracted DNA polymerase inhibitors.

Building on previous studies of cytosine methylation in aDNA, we report the detection of cytosine methylation via direct bisulfite sequencing in human skeletal remains from all five geographic localities studied. The aDNA samples selected for this study previously amplified both nuclear and mitochondrial loci [23–25], demonstrating that both sources of aDNA are well preserved in these samples. This study presents evidence of cytosine methylation in a large collection of human remains, demonstrating that it is possible to analyze epigenetic patterns in ancient populations using direct bisulfite sequencing approaches.

## Materials and Methods

### DNA Samples

We selected 30 samples from five distinct localities throughout North America for analysis: Indian Knoll in west central Kentucky, Ricketts Mound in eastern Kentucky, Klunk Mound in western Illinois, the Yukisma site in northern California, and Xaltocan in central Mexico (Table 1). The archaeological contexts of these localities range in age from approximately 200 ybp to more than 4500 ybp, and locations of the sampled sites have been described in more detail in previous publications [23–25]. Ancient DNA extracts were previously obtained from Native American skeletal remains from these sites, and earlier analyses demonstrated the preservation of endogenous aDNA through the successful, repeated, and independently verified amplification of both mitochondrial and nuclear loci [23–25].

**Table 1:** Provenience and Specifications for the Ancient Human Samples.

Sample ID	Extraction method	Locality	Cultural Affiliation	Site Date	Reference
**OH93**	Silica, GITC	Indian Knoll, Kentucky	Green River	4050–2550 BCE	Bolnick and Bonine, unpublished data
**OH98**	Silica, GITC	Indian Knoll, Kentucky	Green River	4050–2550 BCE	Bolnick and Bonine, unpublished data
**OH266**	Silica, GITC	Indian Knoll, Kentucky	Green River	4050–2550 BCE	Bolnick and Bonine, unpublished data
**OH298**	Silica, GITC	Indian Knoll, Kentucky	Green River	4050–2550 BCE	Bolnick and Bonine, unpublished data
**RIC6**	Phenol-Chloroform	Rickets Mound, Kentucky	Adena	550 BCE -150 CE	Bolnick and Bonine, unpublished data
**RIC10**	Phenol-Chloroform	Rickets Mound, Kentucky	Adena	550 BCE -150 CE	Bolnick and Bonine, unpublished data
**RIC13**	Phenol-Chloroform	Rickets Mound, Kentucky	Adena	550 BCE -150 CE	Bolnick and Bonine, unpublished data
**RIC14**	Phenol-Chloroform	Rickets Mound, Kentucky	Adena	550 BCE -150 CE	Bolnick and Bonine, unpublished data
**RIC15**	Phenol-Chloroform	Rickets Mound, Kentucky	Adena	550 BCE -150 CE	Bolnick and Bonine, unpublished data
**C34-1**	Phenol-Chloroform	Pete Klunk Mound Group, Illinois	Middle Woodland (Hopewell)	175 CE	Bolnick and Smith 2007
**C34-15**	Phenol-Chloroform	Pete Klunk Mound Group, Illinois	Middle Woodland (Hopewell)	175 CE	Bolnick and Smith 2007
**C34-24**	Phenol-Chloroform	Pete Klunk Mound Group, Illinois	Middle Woodland (Hopewell)	175 CE	Bolnick and Smith 2007
**C34-29**	Phenol-Chloroform	Pete Klunk Mound Group, Illinois	Middle Woodland (Hopewell)	175 CE	Bolnick and Smith 2007
**C35-18**	Phenol-Chloroform	Pete Klunk Mound Group, Illinois	Middle Woodland (Hopewell)	175 CE	Bolnick and Smith 2007
**C40-59**	Phenol-Chloroform	Pete Klunk Mound Group, Illinois	Middle Woodland (Hopewell)	175 CE	Bolnick and Smith 2007
**B23**	Silica, GITC	Yukisma site, California	Muwekma Ohlone	1210–1720 CE	Villanea et al. 2013
**B43**	Silica, GITC	Yukisma site, California	Muwekma Ohlone	1210–1720 CE	Villanea et al. 2013
**B50**	Silica, GITC	Yukisma site, California	Muwekma Ohlone	1210–1720 CE	Villanea et al. 2013
**B134**	Silica, GITC	Yukisma site, California	Muwekma Ohlone	1210–1720 CE	Villanea et al. 2013
**B170**	Silica, GITC	Yukisma site, California	Muwekma Ohlone	1210–1720 CE	Villanea et al. 2013
**Y2.2**	Silica, GITC	Xaltocan, Mexico	Otomi*	1240–1360 CE	Mata-Míguez et al. 2012
**Y2.3**	Silica, GITC	Xaltocan, Mexico	Otomi*	1240–1360 CE	Mata-Míguez et al. 2012
**Y2.5**	Silica, GITC	Xaltocan, Mexico	Otomi*	1240–1360 CE	Mata-Míguez et al. 2012
**Y2.7**	Silica, GITC	Xaltocan, Mexico	Otomi*	1240–1360 CE	Mata-Míguez et al. 2012
**Y3.4**	Silica, GITC	Xaltocan, Mexico	Otomi*	1240–1360 CE	Mata-Míguez et al. 2012
**Y3.9**	Silica, GITC	Xaltocan, Mexico	Otomi*	1240–1360 CE	Mata-Míguez et al. 2012
**E10.2**	Silica, GITC	Xaltocan, Mexico	Tepanec/Aztec	1390–1520 CE	Mata-Míguez et al. 2012
**E30.A**	Silica, GITC	Xaltocan, Mexico	Tepanec/Aztec	1390–1520 CE	Mata-Míguez et al. 2012
**E5.1**	Silica, GITC	Xaltocan, Mexico	Tepanec/Aztec	1390–1520 CE	Mata-Míguez et al. 2012
**E8.5**	Silica, GITC	Xaltocan, Mexico	Tepanec/Aztec	1390–1520 CE	Mata-Míguez et al. 2012

GITC: Guanidinium thiocyanate.

*Probable linguistic affiliation based on archaeological inference

Because many of these ancient samples were from burials that are culturally unaffiliated under the Native American Graves Protection and Repatriation Act (NAGPRA), approval for genetic research with these remains was granted by the museums and institutions that curate the collections (Indian Knoll and Ricketts Mound: William S. Webb Museum of Anthropology at the University of Kentucky; Klunk Mound: Bioanthropology Laboratory at Indiana University, under the supervision of Della Collins Cook). Ancient DNA from the Yukisma site, an ancestral burial ground affiliated with the Muwekma Ohlone Tribe, was analyzed with approval from the Muwekma Ohlone Tribal Council, which provided a written letter of support for this research. Collections from the Yukisma site are curated at Washington State University under the supervision of Brian Kemp. Finally, the Instituto Nacional de Antropología e Historia (INAH), which oversees research involving archaeological collections of human skeletal remains in Mexico, provided written permission for the skeletal samples from Xaltocan to be exported for analysis. Xaltocan community leaders also provided oral consent and expressed their support for ancient DNA analyses of the pre-Hispanic Xaltocan burials during consultations with the Xaltocan town council. The skeletal collections from Xaltocan are curated at the Delegaciόn of Xaltocan.

To contextualize the aDNA methylation data and to help identify any differences in methylation status among the ancient samples that might be due to post-mortem DNA degradation, we collected buccal swabs from seven living individuals with written informed consent and extracted DNA using Qiagen’s Blood and Tissue Kit. For an additional, tissue-specific comparison, we also extracted DNA from bone samples of five individuals who died in the last 4–5 years, following the protocol described in [26]. These bone samples were provided by the Forensic Anthropology Research Facility at Texas State University (FACTS), where they are curated under the supervision of Daniel Wescott and Deborah Cunningham. Human remains curated at FACTS are acquired through the Texas Anatomical Gifts Act with written informed consent. The extraction and analysis of DNA from ancient skeletal and contemporary buccal material in this study was also approved by the University of Texas at Austin Institutional Review Board (protocol #2012-05-0105).

### Bisulfite Conversion

We prepared aliquots of the DNA extracts for methylation analysis using the EpiTect Bisulfite Kit (Qiagen) following the manufacturer’s protocol for converting unmethylated cytosines in small amounts of fragmented DNA. This protocol converts unmethylated cytosines to uracils by treatment with sodium bisulfite (NaHSO_3_). Uracils are subsequently incorporated as thymines during target amplification, so any cytosines detected during sequencing are methylated cytosines, which are resistant to sodium bisulfite conversion. We modified the standard protocol by UV irradiating buffers BL (31 mL), BW (13 mL concentrate), BD (3 mL concentrate), and EB (15 mL) for 15 minutes before use in an effort to chemically cross-link and prevent PCR amplification of any DNA contaminants that might be present in these reagents. Five μL of DNA were used in 140 μL bisulfite conversion reactions.

### DNA Amplification, Sequencing, and Analysis

Primers for PCR amplification and pyrosequencing of L1Hs56 in bisulfite converted DNA were designed using the Qiagen Q24 PyroMark Assay Design Software, version 2.0. The primers amplify an 87 base pair (bp) fragment of L1Hs56 (forward primer: 5’-AGTAAAGTTTTTAAGAAATATGGGATTATG, biotinylated reverse primer: 5’-biotin- TTCCATTCTCCACATCACTTTCAAATAC). We prepared PCRs with 2 μL of bisulfite product in a 15 μL reaction volume using Qiagen’s PyroMark PCR kit, and included 0.78 μL of 20 mg/mL BSA (Roche) and 1.5 μL of MasterAmp 10X PCR enhancer with betaine (Epicentre) per reaction. PCR conditions included an initial denaturation at 95°C for 15 minutes, 58 cycles of denaturation at 94°C for 30 seconds, annealing at 58°C for 30 seconds, and extension at 72°C for 30 seconds, and a final extension at 72°C for 10 minutes.

Following amplification, we visualized 3 μL of the amplicons using GelRed on a 6% polyacrylamide gel to confirm amplification prior to pyrosequencing. The remaining PCR product of each confirmed amplicon was submitted to the DNA Sequencing Facility at the University of Texas at Austin for pyrosequencing and CpG analysis on the Qiagen Q24 platform. Pyrosequencing reactions were initiated with a sequencing primer (5’ATATGGGATTATGTGAAAAG) that targets an internal fragment of the 87 bp amplicon and generates a 16 bp read containing a single CpG site. The C:T ratio at the target CpG position was used to calculate percent methylation values for each run, where Cs represent the proportion of methylated cytosines and Ts represent the proportion of unmethylated cytosines following bisulfite conversion.

### DNA Quantitation

To evaluate whether the observed variance in percent methylation values for a given sample was correlated with DNA concentration in the original (non-bisulfite-converted) extracts, we quantitated 16 of the aDNA extracts for a 64 bp fragment of ABO exon 7 (ABO7) using primers described in [27]. Since the L1Hs56 primers are bisulfite specific, they cannot be used to amplify or quantify unconverted DNA. While the abundance of 64 bp and 87 bp fragments presumably differ in our samples, the ABO7 fragment serves as a suitable proxy, allowing us to determine the concentration of small fragments in the original, unconverted aDNA extracts. The aDNA samples selected for this analysis had percent methylation data from at least two independent pyrosequencing runs, from which variance in percent methylation could be calculated.

We prepared quantitative PCRs (qPCRs) with 2 μL of unconverted aDNA extract in a 10 μL reaction volume using the SYBR Fast qPCR kit (Kappa Biosystems), including 1 μL of 20 mg/mL BSA (Roche). qPCRs were performed on the Mastercycler ep realplex qPCR platform with the following conditions: initial denaturation at 95°C for 3 minutes, 50 cycles of denaturation at 95°C for 3 seconds, annealing at 59°C for 30 seconds, and extension at 55°C for 10 seconds, and a 0.5°C/second ramp to 95°C, where runs were held for 15 seconds. qPCR runs were concluded with a melting curve to monitor for possible primer dimers. We included five standardized concentration control reactions to construct a standard curve for quantitation of the 16 samples of unknown concentration. The standard controls contained human genomic DNA prepared at 0.01, 0.05, 0.1, 0.5, and 1 ng/μL. Finally, we included a set of no-DNA template controls to monitor contamination.

Samples with unknown concentrations were prepared in duplicate while standards were prepared in triplicate, and concentrations determined by qPCR were averaged between replicates. Samples with very low concentrations of DNA (below the 0.01 ng/μL standard control) were determined by extrapolation from the standard curve, while all other samples were determined by interpolation. While interpolation of unknowns is ideal, standards prepared below the 0.01 ng/μL level showed inconsistent, stochastic amplification patterns, and were not included in analyses of ABO7 concentration.

### Polymerase Inhibition Analysis

Differences in qPCR measurements of aDNA concentration could be artifacts generated by the presence of inhibitory compounds that are commonly co-extracted with aDNA, which act to decrease DNA polymerase activity [28–30]. If inhibitors substantially interfered with DNA amplification in our samples, then DNA concentration values and subsequent correlations with DNA methylation data would be skewed. To test for the presence of inhibitors, we performed additional rounds of qPCR using an internal positive control (IPC) approach following [28] and [29]. For this assay, we prepared a set of reactions using non-human primate DNA extracted from *Propithecus verreauxi* (Verreaux's sifaka) ear punches, collected by Rebecca Lewis (University of Texas at Austin). We quantified a Short Tandem Repeat (STR) locus, with alleles ranging in size from 109–130 bp, using the species specific primer set 47HDZ22 [31]. Using the SYBR Fast qPCR kit (Kappa Biosystems), including 1 μL of 20 mg/mL BSA (Roche), a set of standards was prepared in duplicate using 2 μL of Verreaux's sifaka DNA in a 10 μL reaction volume. Identical IPC reactions were also prepared in duplicate for each of the 16 aDNA extracts which had previously been assayed for ABO7 concentration, where 1 μL of each aDNA extract was spiked into sifaka reactions to achieve a final concentration of 10%. Finally, we included a set of no-DNA template controls in duplicate to monitor contamination. All qPCRs were performed on the Mastercycler ep realplex qPCR platform with the following conditions: initial denaturation at 95°C for 3 minutes, 40 cycles of denaturation at 95°C for 3 seconds, annealing at 60°C for 30 seconds, and extension at 55°C for 10 seconds, and finally, a 0.5°C/second ramp to 95°C, where runs were held for 15 seconds. qPCR runs were concluded with a melting curve to monitor for possible primer dimers. C_q_ (quantification cycles) values were averaged between replicates, and shifts in C_q_ (ΔC_q_) were calculated as the percent difference between standard and IPC reactions.

### Authentication of aDNA Results

The analysis of highly degraded DNA from ancient human remains requires strict precautions to minimize contamination from exogenous sources of DNA and verify results [22,32–35]. To this end, we performed bisulfite conversion of aDNA extracts and PCR setups in the aDNA facility at the University of Texas at Austin. The aDNA facility is a restricted-access, positive air pressure, HEPA-filter ventilated space with overhead UV-irradiating lights that is dedicated to pre-PCR analyses of aDNA. The post-PCR facility is located in a separate building, and all movement of materials and personnel was unidirectional (from pre-PCR to post-PCR facility) to prevent contamination from DNA amplicons. Additional precautions included the use of sterile and disposable hooded coveralls, hair covers, face masks, sleeve covers, dedicated shoes, and two pairs of gloves. We frequently decontaminated laboratory benchtops and equipment with 6% sodium hypochlorite (full strength bleach), and decontaminated the entire lab space weekly with a 3% sodium hypochlorite solution (1:1::bleach:water, v/v). We also irradiated the facility with a 254-nm emitting UV light for 12 hours following each use, while tubes, containers, and reagents were UV irradiated (when possible) in a 254-nm emitting DNA cross-linker for 15 minutes prior to use.

Negative controls included conversion blanks (bisulfite conversion reaction mixtures containing no DNA) and PCR and qPCR amplification negatives to identify the presence of any contamination at each stage of sample analysis. We performed at least two independent PCR amplifications for each DNA extract, and additional PCR amplifications were performed using an independent DNA extraction from the same individual when sufficient material was available (i.e., for all but eight samples), to verify the authenticity of the results.

During pyrosequencing, the dispensation program included a bisulfite control position to monitor the efficiency of bisulfite conversion in each individual sample. However, because pyrosequencing primers are already designed to amplify bisulfite converted sequences, bisulfite products may be preferentially amplified over unconverted DNA, limiting the accuracy of bisulfite control dispensation as a measure of conversion efficiency. To better evaluate the efficiency of the bisulfite conversion process, eight aDNA samples from four populations were bisulfite converted a second time to assess the reproducibility of the L1Hs56 methylation assay across independent conversions.

Finally, percent methylation results for the Yukisma site samples were independently verified at the Kemp Lab of Molecular Anthropology and aDNA at Washington State University. Ancient DNA was extracted from samples of the same five individuals [28] and bisulfite converted following the protocols described above. PCR products were sent to the University of Texas at Austin DNA Sequencing Facility for pyrosequencing and CpG analysis.

### Statistical Analyses

All statistical analyses were performed using the [R] statistical environment [36]. We eliminated statistical outliers from the percent methylation data when they exceeded two standard deviations of the average percent methylation in the entire dataset, unless they were required for replication purposes (as described in the authentication criteria above). Overall, 5.6% of the methylation data were eliminated from further statistical analysis. In addition to being statistical outliers, these data are thought to result from errors during pyrosequencing because although they passed initial quality checks, several other samples within the same run did not pass and were repeated. Importantly, statistical outcomes are similar with or without outliers included, so the removal of these outliers does not change the overall results or implications of this research. To measure the bisulfite conversion efficiency and precision of the methylation assay (i.e. consistency of percent methylation values between independent replicates for a given sample), we performed student’s *t*-tests using the percent methylation data for eight samples. For these samples, percent methylation data was collected from two independent bisulfite conversions of a single extraction.

Because time since death and depositional conditions are known to influence aDNA preservation [16, 22], we assessed their effects on our measures of cytosine methylation by performing analyses of variance (ANOVAs) in the [R] statistical environment. The residuals of each linear model did not deviate significantly from normality, so ANOVA assumptions of residual normality were not violated and parametric tests were appropriate.

For the first three ANOVAs, we averaged the percent methylation values for each individual sample across all technical replicates of all extracts and secondary bisulfite conversions. In the first ANOVA, we grouped samples by locality and compared mean percent methylation values to assess the influence of geographic location and potential differences in depositional conditions on methylation signal. Second, to evaluate whether percent methylation changes over time, we grouped samples from the various sites into three periods based on the available radiocarbon dates for the archaeological sites. Third, we grouped all ancient samples together and compared them against the forensic bone samples and the contemporary buccal samples to evaluate how methylation data from degraded aDNA compared with a more well-preserved, tissue-specific sample, as well as to an un-degraded (buccal) sample.

To further evaluate the relationship between tissue specific measures of methylation, we compared a subset of ancient samples to the forensic samples via ANOVA. We limited this comparison to samples with variances in methylation falling within two standard deviations of the data generated from the forensic samples. The means in percent methylation from the resulting six aDNA samples were compared to the means in percent methylation from the remaining four forensic samples.

To assess whether the dispersion of percent methylation values observed for a given sample was affected by time or locality, we performed an additional set of ANOVAs based on the range in percent methylation for each sample (i.e., maximum %—minimum % observed). As before, we ran ANOVAs with the data grouped by geographic locality, archaeological time period, or ancient vs. contemporary cohorts to test for possible effects of location and time.

Finally, we investigated whether the variance in percent methylation values for a given sample was correlated with DNA concentration or the presence of DNA polymerase inhibitors in 16 aDNA samples. We calculated the variance in percent methylation for each sample, then used linear regressions in [R] to assess how these variances relate to aDNA concentration and measures of inhibition.

## Results

Of the 30 aDNA samples examined in this study (all of which previously yielded mtDNA and nuclear DNA sequences confirmed through multiple independent analyses), all produced at least one amplicon of L1Hs56 from which percent methylation could be determined (Table 2; S1 Fig). In one sample from the Klunk Mound Group (C34-29), we obtained percent methylation data from only a single amplicon, and were unable to replicate this result through additional PCRs or subsequent bisulfite conversions of the original sample. For the remaining 29 samples, we were able to amplify L1Hs56 repeatedly, and determined percent methylation for each successful amplicon. In total, 29 samples (97%) yielded reproducible amplicons from the same aDNA extract. Secondary aDNA extracts were available for 22 of these 29 samples, and we successfully amplified L1Hs56 from both extracts in all cases. Percent methylation at the variable CpG position in the 87 bp L1Hs56 amplicon ranged between 37% and 78% in the ancient samples, with an average value of 53% methylation (Table 2).

**Table 2:** Percent Methylation of L1Hs56 for Ancient Human Samples.

Sample	Locality	Date	Extract	Bisulfite Conversion 1			Bisulfite Conversion 2	
				PCR 1	PCR 2	PCR3	PCR 1	PCR 2
OH93	Indian Knoll, Kentucky	4050–2550 BCE	1	65	57	44		
OH98	Indian Knoll, Kentucky	4050–2550 BCE	1	50	58		54	54
OH266	Indian Knoll, Kentucky	4050–2550 BCE	1	37	48	46		
OH298	Indian Knoll, Kentucky	4050–2550 BCE	1*	55	58	48		
			1*	63	65			
RIC6	Ricketts Mound, Kentucky	550 BCE -150 CE	1	69	60			
			2	70				
RIC10	Ricketts Mound, Kentucky	550 BCE -150 CE	1	46				
			2	55	52			
RIC13	Ricketts Mound, Kentucky	550 BCE -150 CE	1	51	50			
RIC14	Ricketts Mound, Kentucky	550 BCE -150 CE	1	47	41			
			2	50	52			
RIC15	Ricketts Mound, Kentucky	550 BCE -150 CE	1	50	51			
			2	44	50			
C34-1	Pete Klunk Mound, Illinois	175 CE	1	60	47			
			2	50				
C34-15	Pete Klunk Mound, Illinois	175 CE	1	50	50			
			2	60				
C34-24	Pete Klunk Mound, Illinois	175 CE	1	70	46	47		
C34-29	Pete Klunk Mound, Illinois	175 CE	1	48				
C35-18	Pete Klunk Mound, Illinois	175 CE	1	77	71			
			2	69				
C40-59	Pete Klunk Mound, Illinois	175 CE	1	47	45		50	47
			2	45	47			
B23	Yukisma site, California	1210–1720 CE	1	49	50		47	45
B43	Yukisma site, California	1210–1720 CE	1	50	49		54	54
			2	78				
B50	Yukisma site, California	1210–1720 CE	1	50	47		51	48
			2	58	64			
B134	Yukisma site, California	1210–1720 CE	1	53	50		52	48
			2	61				
B170	Yukisma site, California	1210–1720 CE	1	53	52		49	50
			2	57				
Y2.2	Xaltocan, Mexico	1240–1360 CE	1	44				
			2	46	45		41	42
Y2.3	Xaltocan, Mexico	1240–1360 CE	1	65	66			
			2	55	44	52		
Y2.5	Xaltocan, Mexico	1240–1360 CE	1	76				
			2	48	40			
Y2.7	Xaltocan, Mexico	1240–1360 CE	1	51	60			
			2	53	51			
Y3.4	Xaltocan, Mexico	1240–1360 CE	1	43	43		48	44
			2	49				
Y3.9	Xaltocan, Mexico	1240–1360 CE	1	70				
			2	55	50	55		
E10.2	Xaltocan, Mexico	1390–1520 CE	1	52	54			
			2	51	55			
E30.A	Xaltocan, Mexico	1390–1520 CE	1	46				
			2*	42	53	48		
			2*	56				
E5.1	Xaltocan, Mexico	1390–1520 CE	1	47				
			2	48	60			
E8.5	Xaltocan, Mexico	1390–1520 CE	1	59	63			
			2	56	55			

*In cases where more than three PCR amplifications were performed, data from the same extract are reported in more than one row.

The seven contemporary samples extracted from buccal swabs all amplified the L1Hs56 fragment in three replicate PCRs, and percent methylation ranged between 50% and 64% for these samples, with an average value of 57% (Table 3). The five forensic bone samples also amplified the L1Hs56 fragment in three replicate PCRs, with percent methylation values ranging between 45% and 56%, with an average value of 51% (Table 3).

**Table 3:** Percent Methylation of L1Hs56 in Contemporary Human Buccal Samples and Forensic Bone Samples.

Sample		% Methylation		
		PCR 1	PCR 2	PCR3
*Contemporary Buccal*	1	61	60	50
	2	61	58	62
	3	61	60	64
	4	50	54	50
	5	56	55	56
	6	52	50	52
	7	57	60	58
*Forensic Bone*	D59	51	54	50
	D70	50	53	56
	D99	51	45	46
	D10	52	55	52
	D29	52	49	51

Pyrosequencing controls indicated that both the bisulfite conversion process and the pyrosequencing reactions performed as expected. The pyrograms used in the final analyses showed no cytosine misincorporations in the bisulfite control positions (S1A Fig, dispensation 4), and control dispensations (S1A Fig, dispensation 7) indicated negligible levels of background noise in all pyrosequencing reactions. All negative controls, including both bisulfite conversion blanks and the PCR blanks, showed no amplification, demonstrating that no exogenous DNA contamination was introduced during bisulfite conversion or PCR setup (S1B Fig). Furthermore, when we compared percent methylation values from independent bisulfite conversions of the same aDNA extracts, no statistically significant differences were observed (*P* ≥ 0.07, Table 4). These results suggest that the bisulfite conversion process was efficient and that percent methylation results were reproducible across bisulfite conversions.

**Table 4:** Results of Student’s *t*-Test between Independent Bisulfite Conversions of 8 aDNA Samples.

Sample	Site	*P*-value
WSU23	Yukisma site	0.26
WSU43	Yukisma site	0.07
WSU134	Yukisma site	0.20
WSU170	Yukisma site	0.20
K1159	Klunk Mound Group	0.13
OH98	Indian Knoll	1.00
Y222	Xaltocan	0.16
Y341	Xaltocan	0.37

**Fig 2:**
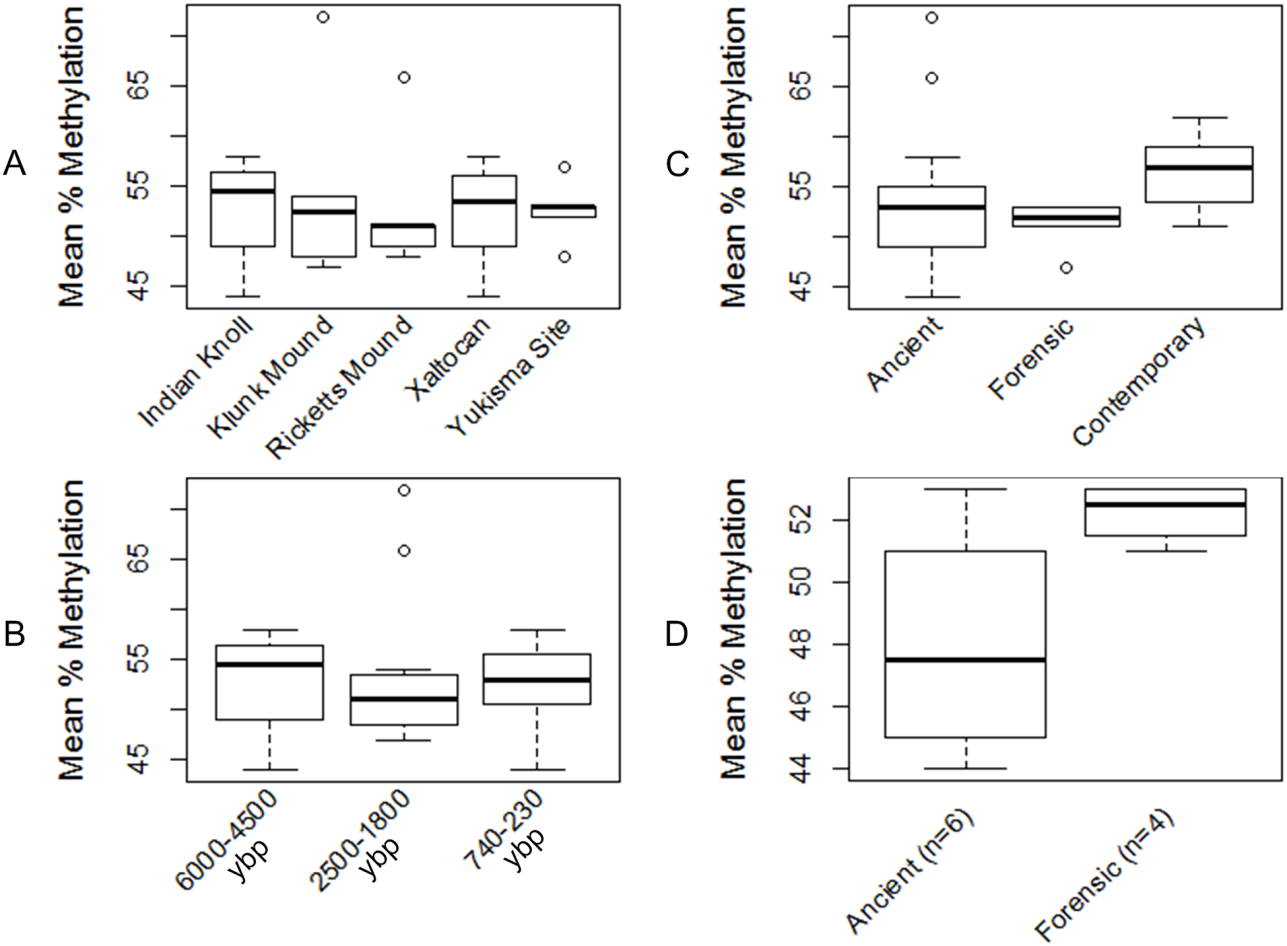
ANOVAs comparing mean % methylation. Mean % methylation is compared (*A*) by locality, (*B*) between three archaeological time periods, (*C*) between ancient, forensic, and contemporary cohorts, and (*D*) between the best performing ancient samples (i.e., with the lowest variance in percent methylation) and forensic samples.

Despite the efficiency of this L1Hs56 bisulfite sequencing assay, percent methylation values varied from sample to sample (Table 2). Because time since death and depositional conditions are known to affect aDNA preservation [16, 22], we conducted a series of one-way ANOVAs to assess whether time or geographic locality influenced this variability. At some locations, such as Indian Knoll, Klunk, and Xaltocan, mean percent methylation varied substantially between samples from the same site; at other locations, such as the Yukisma and Ricketts sites, less variation was evident. However, because the ranges of variation overlapped across sites, the ANOVA indicated no statistically significant differences (*P* = 0.98) in mean percent methylation among the five localities studied (Fig 2A, Table 5). When the ancient samples were divided into three separate time periods (Fig 2B), we again observed no significant differences (*P* = 0.87) in mean percent methylation (Table 5). Finally, when all ancient samples were grouped together and compared with the forensic bone samples and buccal samples from contemporary individuals, we observed a lower average percent methylation in the ancient and forensic samples (Fig 2C), but this difference was not statistically significant (*P* = 0.22; Table 5). Thus, while mean percent methylation varied from sample to sample, we found no clear, statistically significant patterning to this variability according to locality or time.

**Table 5:** Analysis of Variance (ANOVA) Results.

	Categorical Variables	df	SS	MS	F	*P*-value
Mean % Methylation	Geographic Locality	4	15.3	3.82	0.095	0.98
	Archaeological Period	2	10.3	5.15	0.138	0.87
	Ancient vs. Forensic vs. Contemporary	2	93.9	46.93	1.589	0.22
	Ancient (n = 6) vs. Forensic (n = 4) Subsets	1	43.4	43.35	5.527	**0.05**
Dispersion (Range) in % Methylation	Geographic Locality	4	198.2	49.55	0.711	0.59
	Archaeological Period	2	180.9	90.44	1.388	0.27
	Ancient vs. Forensic vs. Contemporary	2	532.9	266.44	5.166	**0.01**

df: degrees of freedom; SS: sum of squares; MS: mean square

To assess how the best performing ancient samples (i.e. those with the lowest variance in percent methylation) compared with less degraded DNA derived from bone, we restricted the ANOVA to the set of ancient samples with methylation data that fell within two standard deviations of the forensic samples. In this analysis, we observed that the ancient samples had a wider variance and lower average percent methylation than the younger forensic samples (*P* = 0.05; Fig 2D, Table 5). This result indicates that the ancient samples with the lowest variance in percent methylation were significantly less methylated than the more recent forensic bone samples.

**Fig 3:**
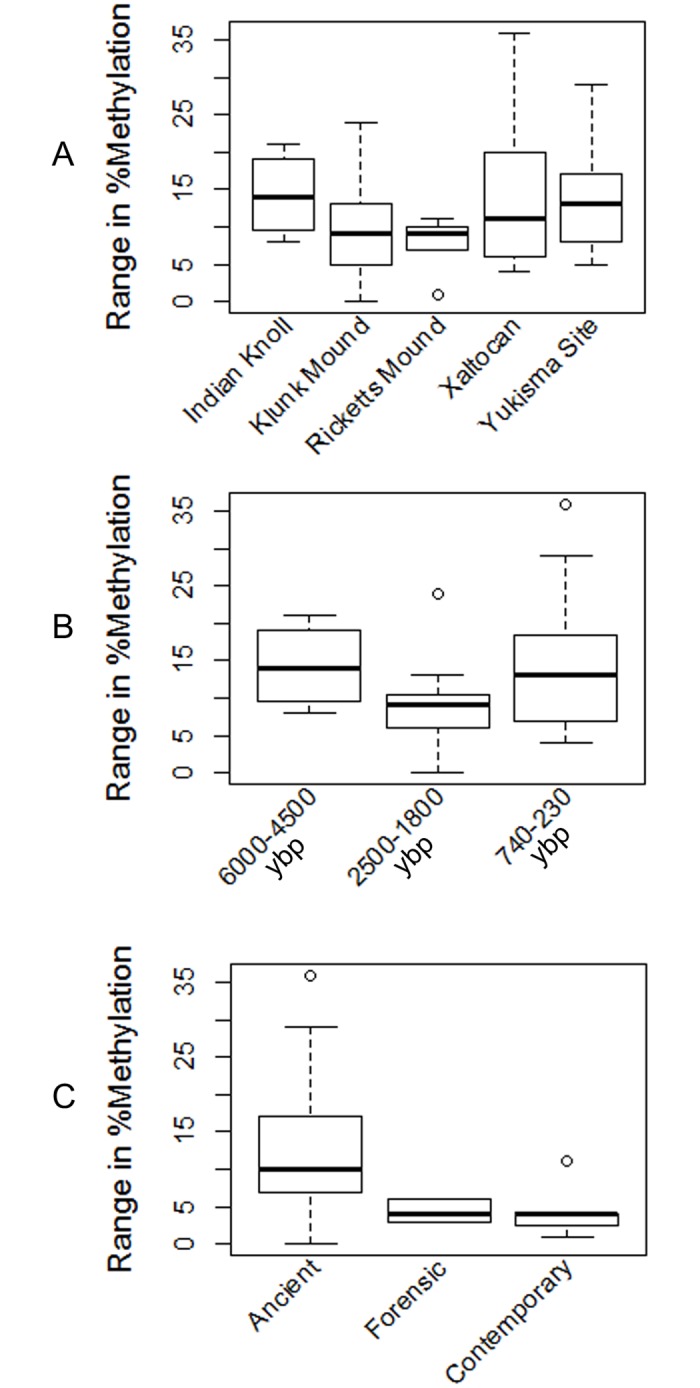
ANOVAs comparing dispersion (range) in % methylation. Dispersion in % methylation is compared (*A*) by locality, (*B*) between three archaeological time periods, and (*C*) between ancient, forensic, and contemporary cohorts.

We also observed variation between samples in the dispersion (range) of percent methylation values for a given sample. Some samples performed consistently and yielded similar values of methylation across runs, whereas other samples varied much more widely from run to run. For example, percent methylation results for Xaltocan sample E10.2 ranged from 51–55%, while results for Xaltocan sample Y2.5 ranged from 40–76%. To assess whether the degree of dispersion for each sample was affected by locality or time, we performed a second round of ANOVAs (Fig 3, Table 5). No significant differences were detected when samples were compared by locality (Fig 3A, *P* = 0.59), indicating that locality cannot explain the variation in dispersion of percent methylation values for each sample. Nor did we observe any significant differences in degree of dispersion between archaeological time periods (Fig 3B, *P* = 0.27). However, when we grouped all ancient samples together and compared them with the contemporary and forensic samples, we found that the ancient samples exhibited significantly greater dispersion in the percent methylation values for each sample than either the forensic or contemporary samples (Fig 3C, *P* = 0.01).

**Fig 4:**
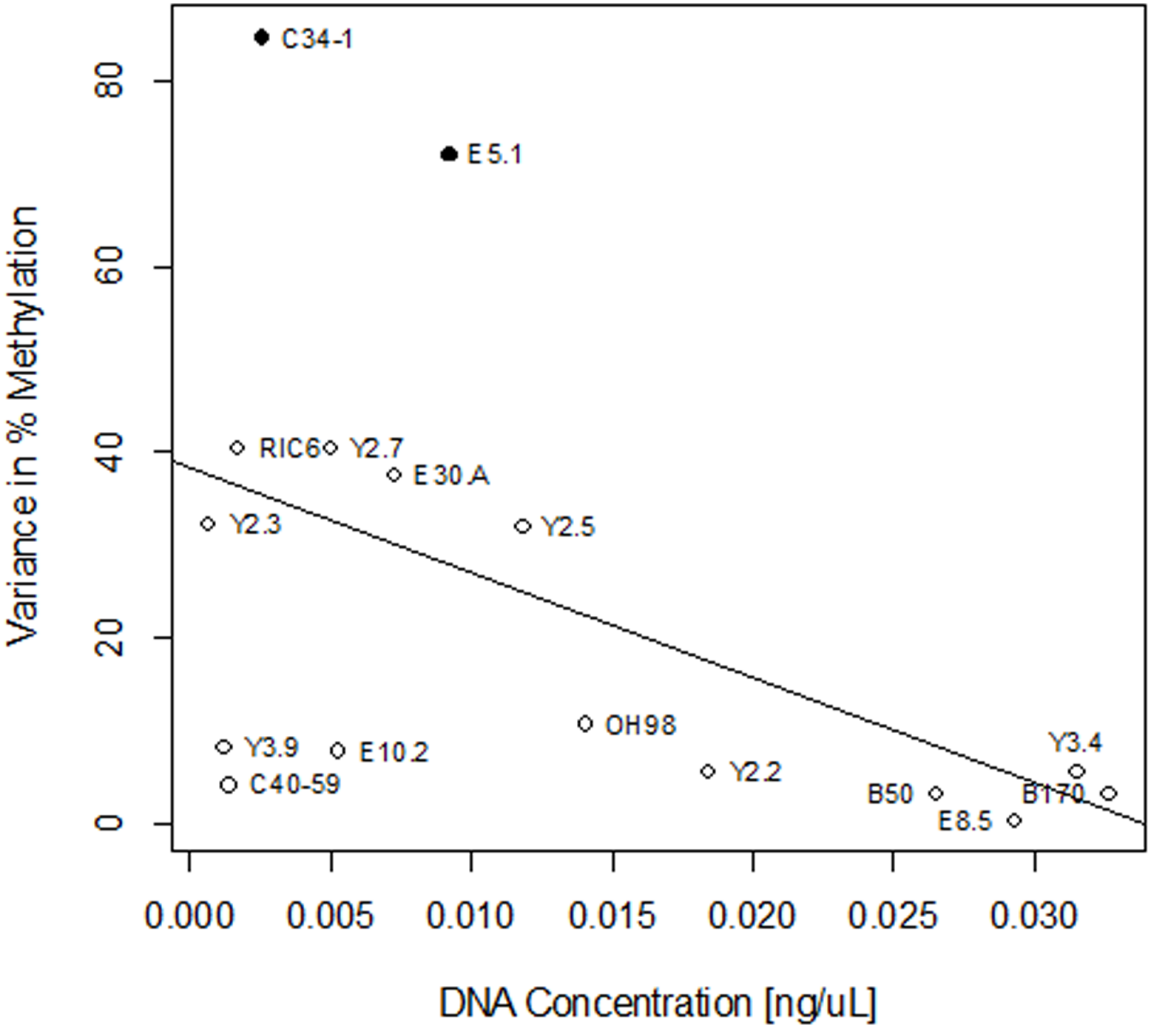
Linear Regression of Variance in % Methylation as a Function of aDNA Concentration. Samples which showed high levels of PCR inhibition are indicated by solid black circles.

These findings suggest that DNA degradation may increase the variability in percent methylation reads for a sample, but the consequences of DNA degradation may not be strictly determined by locality or increasing time since death. We therefore hypothesized that DNA degradation and sample-by-sample variation in DNA preservation may influence the degree of dispersion in percent methylation values for each sample. To test this hypothesis, we determined the DNA concentration of a 64 bp fragment of ABO exon 7 (ABO7) in unconverted material from 16 aDNA extracts via qPCR, and regressed those values against the associated variances in percent methylation for each sample (Table 6). The results showed that variance in percent methylation was negatively correlated with the concentration of aDNA in the original, unconverted extracts (Fig 4, R^2^ = 0.26; *P* = 0.04; df = 15). Thus, samples with higher DNA concentrations showed lower variances in percent methylation than those with lower concentrations of starting material.

**Table 6:** Results of ABO7 qPCR and Internal Positive Control Test for PCR inhibition.

Sample	Extract	Concentration *(ng/μL)*	Variance in % Methylation	Mean C_q_ Cycle	% Difference *(IPC and Sample C*_*q*_ *)*
B170	1	0.0326	3.33	24.67	1.90
B50	1	0.0265	3.33	24.53	1.32
C34-1	1	0.0025	84.50	28.11	**16.11**
C40-59	1	0.0013	4.25	24.91	2.89
E10.2	2	0.0052	8.00	25.25	4.30
E30.A	2	0.0072	37.58	24.44	0.95
E5.1	2	0.0092	72.00	26.71	**10.33**
E8.5	2	0.0293	0.50	24.64	1.78
OH98	1	0.0140	10.67	25.12	3.76
RIC6	1	0.0017	40.5	25.48	5.25
Y2.2	2	0.0184	5.67	24.82	2.52
Y2.3	2	0.0007	32.33	24.49	1.16
Y2.5	2	0.0118	32.00	24.8	2.44
Y2.7	1	0.0050	40.50	24.84	2.60
Y3.4	1	0.0315	5.67	24.55	1.40
Y3.9	2	0.0012	8.33	25.08	3.59
IPC Standard	NA	NA	NA	24.21	NA

*NA*: *Not applicable*; High levels of inhibition are in bold.

Finally, because these results could be influenced by the presence of co-extracted inhibitors in the aDNA extracts, we tested for the presence of inhibitors using an IPC approach. All samples exhibited some inhibition (Table 6), but only two samples, E5.1 and C34-1 showed high levels of inhibition (10.33% and 16.11%, respectively). E5.1 and C34-1 were also the two samples in our analysis with the highest variance in percent methylation, suggesting that high variance might be related to inhibition for these samples. Regression analysis of inhibition against variance did show a statistically significant relationship (R^2^ = 0.62; *P* = 0.0003; df = 15). However, these results were disproportionately skewed by samples with abnormally high inhibition (E5.1 and C34-1). When those samples were removed, the relationship between variance in percent methylation and inhibition was no longer significant (R^2^ = 0.008; *P* = 0.76; df = 13). Notably, when E5.1 and C34-1 were similarly removed from the regression of variance in percent methylation against aDNA concentration, the relationship remained statistically significant (R^2^ = 0.34; *P* = 0.03; df = 13).

## Discussion

This study builds on previous efforts to detect cytosine methylation in aDNA, and shows that methylation is recoverable via bisulfite sequencing in large numbers of ancient human remains. We successfully amplified L1Hs56 and determined percent methylation at a single CpG dinucleotide in samples from five distinct geographic localities spanning a time period of more than 4500 years. Our results indicate that some aDNA samples, while fragmented by post-mortem processes, can maintain sufficient concentrations of DNA for the detection of cytosine methylation via direct bisulfite sequencing. This holds despite the harsh effects of exposure to sodium bisulfite, which can reduce the number of viable molecules by 90% or more [37].

The results in this study are thought to be derived from endogenous ancient DNA because of strict adherence to laboratory precautions and controls, multiple independent verifications of percent methylation data for each sample, and an absence of contaminant DNA in all negative control reactions. We also identified differences in methylation levels between contemporary and ancient samples, indicating that the ancient results are not due to contamination. Furthermore, previous analyses repeatedly demonstrated the presence of endogenous ancient mtDNA and nuclear DNA in the aDNA extracts used in this study [23–25], and showed that contamination from contemporary sources had not been introduced prior to our analysis. However, because this study used standard pyrosequencing, which does not yield data on individual DNA molecules, we were unable to assess DNA damage patterns as a further means of authenticating our results. Another limitation of our study design is that we analyzed a fixed monoallelic locus (to reduce sequence variation in our assay), which meant there are no diagnostic polymorphisms that can be used to identify potential contaminant sequences. Nevertheless, the extensive precautions used to destroy, prevent, and detect contamination in this study, as well as the observed differences between ancient and contemporary methylation levels, indicate that our results are derived from endogenous aDNA.

In this study, we developed an assay for assessing whether cytosine methylation is preserved in human aDNA. Because methylation within single copy loci, such as differentially methylated regions (DMRs) and gene promoters, can vary due to functional differences in epigenetic gene regulation, it may be difficult for aDNA studies to determine whether observed variation in percent methylation for these single copy loci is due to (a) normal variation in gene expression, or (b) confounding factors such as cytosine damage stemming from post-mortem degradation processes. To avoid this pitfall, we targeted a single CpG within a repetitive element (L1Hs56), which is expected to be methylated in healthy, somatic tissues [6,7]. Targeting a locus with a known methylation status reduces the possibility that variation in methylation levels between samples is due to individual differences in gene expression. Instead, an inability to detect cytosine methylation, or variation in detectable levels of methylation between samples, can be more directly associated with issues of DNA preservation, such as post-mortem degradation that results in sequence damage and reduces the number of viable template molecules.

Using this assay, we were able to evaluate how time since death (over a period of 4500 years) and depositional conditions (across five different localities), two factors that significantly influence aDNA preservation [22], may have affected our ability to detect and reconstruct cytosine methylation signals in aDNA. We found that geographic locality had no significant effect and could not account for the variation we observed in percent methylation among our samples. Since we selected samples for analysis that had previously amplified for mtDNA and nuclear DNA, it seems that as long as the environmental conditions at a particular site are suitable for aDNA preservation, cytosine methylation can be detected.

We also found that there is not a simple linear relationship between preservation of cytosine methylation in aDNA and time. The aDNA samples exhibited a somewhat lower mean percent methylation than contemporary buccal samples (although the difference was not statistically significant), and the range (dispersion) of percent methylation values obtained for individual aDNA samples was significantly wider than the range obtained for forensic bone or contemporary buccal samples. These results suggest that post-mortem DNA degradation can influence methylation signal. However, changes in the methylation signal in aDNA do not scale linearly with increasing time, suggesting that most of the relevant post-mortem changes may occur fairly soon after death. This pattern would be consistent with other evidence of a nonlinear relationship between time of death and aDNA degradation, where most post-mortem damage occurs rapidly after death, with subsequent slower rates of degradation over time (given appropriate depositional conditions) ([16, 38–40], but see [41]).

While geographic locality and time since death do not explain the variation we detected between samples, individual sample preservation and post-mortem DNA degradation does matter. DNA concentration was inversely correlated with the variance in percent methylation values for a given sample, indicating that samples with better DNA preservation yielded more consistent signals of percent methylation between independent pyrosequencing runs. While inhibition affected two samples (E5.1 and C34-1) enough that it may have contributed to the higher variance in percent methylation observed in those samples, the variance in methylation observed in the other samples cannot be attributed to the presence of DNA polymerase inhibitors. We note that variability in measures of percent methylation markedly decreased in samples with a DNA concentration above approximately 0.015 ng/μL. Using our assay, samples at or above this concentration produced much more precise measures of cytosine methylation. This may indicate the presence of a minimum threshold concentration, above which aDNA samples are amenable to cytosine methylation analysis via bisulfite sequencing. However, while this concentration serves as a precision threshold for the methylation data in this study, we caution that it should not be considered a definitive standard. Defining such a standard will require further research, and will presumably vary with assay parameters. Nonetheless, our results indicate that given sufficiently well-preserved endogenous DNA and relatively low amounts of co-extracted inhibitors, precise measures of cytosine methylation at a single nucleotide resolution are possible with some aDNA samples.

The higher variability observed in more poorly preserved samples may reflect the stochastic nature of target amplification with low copy number DNA. The CpG site we investigated in the L1Hs56 fragment should occur in both methylated and unmethylated forms in every DNA sample because methylation varies between alleles for this non-regulatory portion of the LINE insertion. In more highly concentrated samples, amplicons should be generated from a greater number of starting template molecules in each PCR. Because the percent methylation value determined during pyrosequencing is averaged over molecules with varying methylation states, PCRs that start from a large number of template molecules will tend to be more representative of the methylation pattern in the DNA extract as a whole. Well-preserved samples should therefore yield fairly consistent percent methylation values between runs. Conversely, in poorly preserved samples with lower DNA concentrations, the smaller number of starting template molecules in each PCR tends to result in a less representative measure of percent methylation and wider swings between runs. Much like allelic dropout, more molecules of a particular methylation state may be synthesized by chance in the early stages of target amplification, obscuring the presence of alternate states in downstream analyses. Thus, percent methylation data from samples with poor DNA preservation should be carefully scrutinized as they are often highly variable, and should not be taken as indicative of the actual methylation state in the living organism. Well preserved samples that generate consistent measures of percent methylation, on the other hand, may yield a more accurate picture of methylation status.

We note that variability in samples with low DNA concentration may introduce different expectations for detection bias in different cytosine methylation contexts. For example, in regions of very high *in vivo* methylation, variability in measures of percent methylation may lead to underestimation biases. In regions of very low *in vivo* methylation, on the other hand, overestimation biases may occur. Since L1Hs56 is not a site of either very high or very low cytosine methylation, specific directional biases for cytosine methylation estimates are not expected. Such biases should be considered, however, in future research investigating cytosine methylation of other loci.

The other trend that we observed, for ancient samples to exhibit lower average methylation than the contemporary buccal and forensic bone samples, also merits discussion. The differences we observed between cheek epithelium and both osseous samples may reflect tissue-specific differences in L1Hs methylation levels. However, while both osseous tissues had more similar levels of L1Hs56 methylation compared to buccal epithelium, this pattern may not stem from tissue specific patterns of methylation, but rather from DNA degradation, as all osseous tissues used here were necessarily collected post-mortem. Nonetheless, when we compared osseous tissues alone and restricted the analysis to the ancient samples with lowest variance in percent methylation (i.e. the samples with higher DNA quality), the ancient samples showed a significantly lower mean methylation than the more contemporary forensic samples. While we have ruled out a simple relationship between time since death and mean percent methylation, it is clear that ancient samples are hypomethylated compared to more contemporary sources of DNA. We consider several possible explanations for this pattern.

First, lower measurements of percent methylation in ancient samples may stem from DNA degradation in the ancient samples. Previous studies of cytosine methylation in ancient plants have shown that deamination of methylated cytosines to thymines is a time-dependent diagenetic process, where methylated cytosines decay exponentially over time [16]. Because deaminated 5mCs (e.g., thymines) are indistinguishable from unmethylated cytosines following bisulfite conversion and PCR amplification, deamination of 5mCs artificially decreases the methylation signal over time from its original status in living organisms when using bisulfite techniques. Thus, older samples may show lower methylation than more contemporary ones. Diagenetic processes may also explain the lower rate of methylation in older samples if decay kinetics differ substantially between methylated and unmethylated cytosines and their degradation products. If methylated cytosines are deaminated at different rates than unmethylated cytosines, or if rates of strand breakage differ between cytosine and its deamination products thymine and uracil, assessments of cytosine methylation could be affected. Finally, because DNA extracted from osteological remains may come from a heterogeneous mix of peripheral blood and bone tissues, tissue specific differences in methylation could explain lower methylation among older samples in this study. Specifically, if L1Hs56 in peripheral blood is methylated to a higher degree than in bone, the decay of peripheral blood tissues from the bone matrix during diagenesis might result in lower methylation among more ancient samples. Further research is necessary to distinguish among these possibilities.

Another possible explanation for the lower methylation signal in the ancient samples stems from the relationship between nutritional intake and genome-wide levels of cytosine methylation. It has recently been shown that a lack of methyl donors in the diet can lead to global genomic hypomethylation, independent of any specific pathology [42]. Thus, if post-mortem damage could be ruled out as a confounding factor, the observed differences in percent methylation between ancient and contemporary samples might be genuine, resulting from underlying differences in nutritional intake between ancient and contemporary individuals.

In the present study, it is unclear if cytosine deamination played a role in decreasing the percent methylation detected among the ancient samples. Deamination is typically associated with points of strand breakage in aDNA, so deaminated cytosines are highly concentrated in overhangs and accumulate there as a time-dependent diagenetic process [43–44]. Since our target CpG was located in the center of the 87 bp portion of the L1Hs56 sequence, PCR amplicons should have been generated primarily from fragments that were intact over the nucleotides immediately surrounding the cytosine of interest. Therefore, given the methods used here, the impact of DNA damage on percent methylation values might not be substantial, and might explain why other studies detected a time-dependent diagenesis of cytosine methylation while we did not. Furthermore, many of the ancient samples produced percent methylation estimates that were as consistent as those from the forensic samples, indicating that they may be no more degraded. The observed differences in percent methylation between sites, and between ancient and contemporary cohorts, might instead represent variability in nutritional status or naturally occurring variation in L1Hs methylation patterns. However, because this study was not designed to distinguish between these potential causes of variation in percent methylation, more research is needed to distinguish among these possible explanations. In particular, future research using aDNA repair protocols, bisulfite conversion, and next generation sequencing approaches would be valuable. Future studies should also investigate whether decreased methylation in the genome is a regional or global phenomenon to help determine whether nutritional difference is a plausible determinant of lower methylation in ancient samples.

While we have shown that cytosine methylation can be detected in human remains, the potential for human epigenome reconstruction in aDNA will ultimately be limited by at least two factors. First, it is clear from this study that sample quality (i.e. DNA concentration and co-extracted inhibitors) has a significant effect on our ability to precisely determine percent methylation by bisulfite sequencing in aDNA samples. Because differences in epigenetic gene regulation can depend on small differences in percent methylation, accurately inferring gene expression in an ancient individual from the direct bisulfite detection of methylated cytosines may be limited to well-preserved samples where methylation levels can be reconstructed with high confidence. Thus, future studies should explore the relationship between DNA concentration and the accurate determination of percent methylation, to allow a standard to be developed for the minimum DNA concentration required for assays that rely on bisulfite detection of cytosine methylation in aDNA. This will be especially important for future studies seeking to precisely reconstruct levels of gene expression in ancient or extinct organisms using bisulfite methodologies. Second, it will be difficult to reconstruct the whole epigenome of an ancient organism due to the limited availability of ancient source materials. Soft tissues, such as skin, are often not preserved in archaeological contexts, and when they are, DNA is less frequently preserved due to diagenetic processes and microbial activities [41]. Thus, epigenome analysis will largely be limited to teeth and bone, which will primarily contain epigenome information relevant to those tissues.

## Conclusions

This study reports the detection of cytosine methylation in ancient human populations using direct bisulfite sequencing and demonstrates that methylation signals are readily recoverable from ancient skeletal material with preserved nuclear DNA spanning a time depth of more than 4500 years. This study also shows that our ability to determine percent methylation precisely in aDNA samples is related to the degree of DNA preservation in the samples. Our results provide the first evidence that cytosine methylation is recoverable in many aDNA samples, demonstrating that it is possible to study epigenetic patterns in populations of ancient or extinct organisms.

Finally, we note that conventional methods for measuring methylation, including bisulfite conversion, have been described as “impracticable” for ancient samples because these methods induce additional damage to already degraded aDNA [9,45]. While this may be a limiting factor for older or more highly degraded remains with poor DNA quality, our results show that bisulfite sequencing can be used to reconstruct DNA methylation in more recent remains with better aDNA preservation. Thus, bisulfite sequencing approaches may be useful for precisely determining cytosine methylation levels in some aDNA samples, providing a means of generating ancient methylomes at single nucleotide resolution.

## Supporting Information

S1 Fig Representative pyrograms for (*A*) an aDNA sample (Ricketts, burial 6) and (*B*) a no-template control. (TIF)
