# Supplementary figures and images for: Detection of Cytosine Methylation in Ancient DNA from Five Native American Populations Using Bisulfite Sequencing
Source: PLoS One. 2015 May 27;10(5):e0125344. doi: 10.1371/journal.pone.0125344 (PMC4445908; doi:10.1371/journal.pone.0125344)

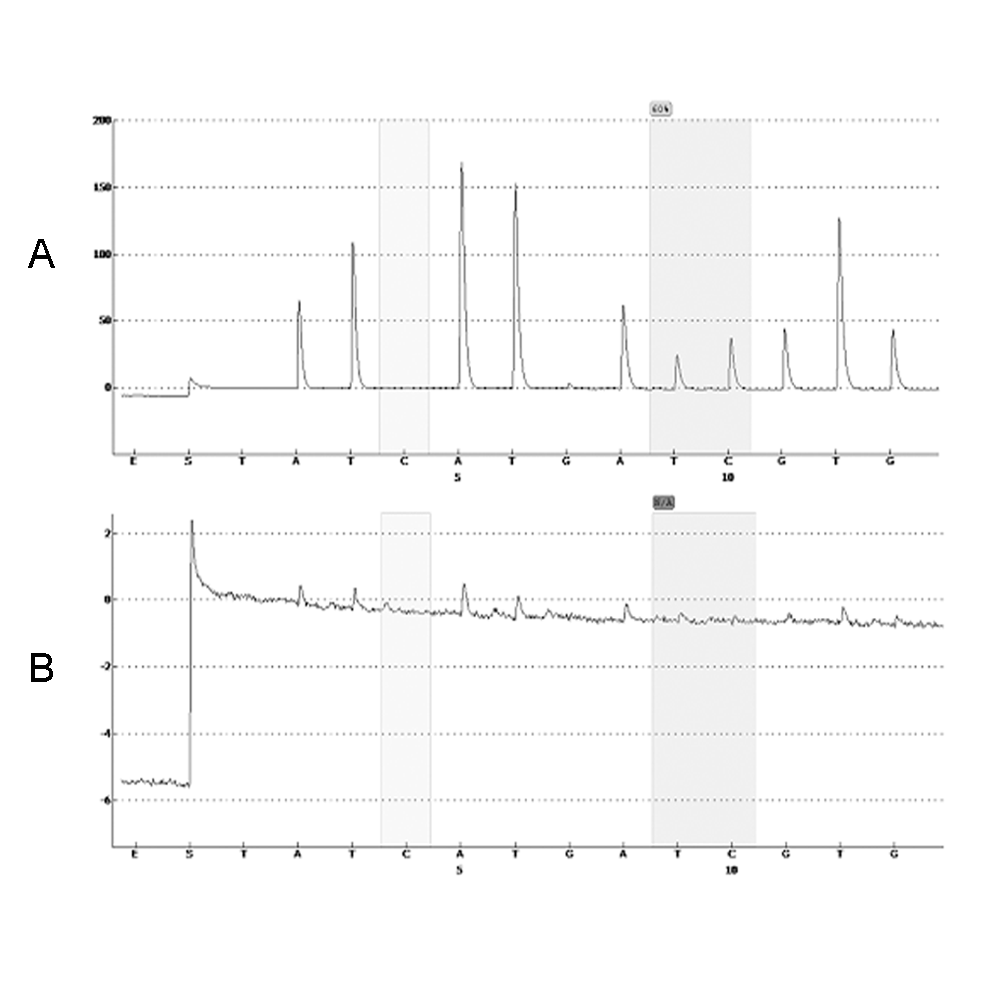

Supplement: S1 Fig — (TIF) [file pone.0125344.s001.tif]
